# Species of the Genus ×*Agrotrigia* (Hordeeae, Poaceae)—A Case of a Different Pattern of Parental rDNA Elimination

**DOI:** 10.3390/plants15162432

**Published:** 2026-08-10

**Authors:** Alexander A. Gnutikov, Nikolai N. Nosov, Inna E. Evnukova, Victoria S. Shneyer, Aleksey V. Troitsky, Alexander V. Rodionov

**Affiliations:** 1 N.I. Vavilov Institute of Plant Genetic Resources (VIR), St. Petersburg 190000, Russia; a.gnutikov@vir.nw.ru; 2 Komarov Botanical Institute (BIN RAS), St. Petersburg 197022, Russia; shneyer@binran.ru (V.S.S.); avrodionov@binran.ru (A.V.R.); 3 Faculty of Biology, St. Petersburg State University, St. Petersburg 199034, Russia; st107716@student.spbu.ru; 4 Belozersky Institute of Physico-Chemical Biology, Lomonosov Moscow State University, Moscow 119992, Russia

**Keywords:** *Agropyron*, *Elytrigia*, grasses, hybridization, ITS, molecular phylogeny, next-generation sequencing

## Abstract

All taxa of plants have undergone several rounds of whole-genome duplication (WGD), accompanying interspecific and intergeneric crosses; subsequently, some part of one or both parent genomes in hybrid plants can be eliminated. The objective of this study was to evaluate the relationships of the intergeneric hybrid ×*Agrotrigia* (Poaceae) species with the taxa of parental genera *Agropyron* and *Elytrigia* and to determine the distribution as well as conservation or loss of parental genomes in the genomes of hybrid species. Molecular phylogenetic analysis of nuclear (ITS, ETS) and several chloroplast DNA markers confirmed that the most likely parents of this hybrid were really species of *Agropyron* and *Elytrigia*. The compositions of ribotypes (sequences of the 18S–ITS1–5.8S rDNA region) assessed by next-generation sequencing (NGS) showed that the genomes of hybrid species of ×*Agrotrigia hajastanica* and of the sample of ×*A. androssovii* from Turkmenistan carry ribotypes of both *Agropyron* and *Elytrigia*. However, the samples of ×*A. kotovii* and ×*A. androssovii* from Pskov Oblast bear ribotypes of the *Elytrigia* type only, whereas the genome of one sample of the ×*Agrotrigia* genus collected in Stavropol Krai consists exclusively of two clusters of *Agropyron*-type ribotypes (P genome) and may represent a new species. Therefore, these two (or three) species may be subject to the elimination of portions of the genome of one of the parents, though the extent of elimination may be different.

## 1. Introduction

According to modern concepts, hybridization is one of the main ways in which flowering plants acquire evolutionary changes [1,2]. All extant plant taxa have undergone several rounds of whole-genome duplication (WGD) resulting from interspecific and even more distant (e.g., intergeneric) crosses [3]. Hybridization often results in the formation of allopolyploid genomes, which allows plants to bypass barriers to sympatric speciation. Complex allopolyploid genomes undergo various genetic processes, such as the loss of all or part of the chromosomes of one parent, transposon expansion, translocations and transpositions, and the loss of part of the genes of one or both parental genomes [3,4,5,6]. Previously, botanists could assume a hybrid origin of a species or an individual plant based on an unusual combination of taxonomically significant traits. Later, hybridogenous species began to be identified via observation of different positions of some taxa relative to each other in phylogenetic trees constructed using chloroplast markers (inherited maternally) and nuclear markers (biparental inheritance) [7]. Now it is possible to identify hybrids and their parents using locus-specific sequencing of multicopy genes on the Illumina platform (next-generation sequencing, NGS), even when the hybrid origin of the organisms is not morphologically obvious.

The objects of our study are representatives of one of the most important families of flowering plants for humans—Poaceae Barnhart. This widespread family includes the tribe Hordeeae Martinov (=Triticeae Dumort.), which contains the particularly valuable genera of cereals *Triticum* L., *Secale* L., and *Hordeum* L. The tribe Hordeeae is of great interest to geneticists and breeders, especially those working with cultivated plants. This tribe is also distinguished in the family for the highest reported frequency of intergeneric hybridization, discovered through karyosystematic analysis. Of course, the well-known food genera of the tribe, as well as hybrids within these groups, have been very popular in molecular phylogenetic and cytogenetic studies [8,9]. We focused on the wild intergeneric hybrid ×*Agrotrigia* Tzvelev. Unusual specimens similar to *Agropyron* Gaertn. and *Elytrigia* Desv. but combining intermediate morphological characteristics of these taxa, mainly of the spike and glumes, deviating more toward one or the other genus, were noted by botanists in their works [10,11,12]. N. N. Tzvelev first expressed the notion that this is a taxonomically independent intergeneric hybrid in his work [13], and the genus ×*Agrotrigia* was formally described [13,14]. Previously, polyploid species from the Hordeeae tribe were divided into several groups by the presence of certain genomes, as determined by cytogenetic markers [15,16]. The most studied genera separated by cytogenetic data are *Pseudoroegneria* (Nevski) Á.Löve (St genome), *Lophopyrum* Á. Löve (E), *Thinopyrum* Á. Löve (J), and *Elymus* L. (StH) [15,16]. The genus *Agropyron* bears P genome [15,16,17,18], whereas *Elytrigia* is a taxonomically complicated taxon and is now divided into different genera according to its genomic structure [17,19]. Our molecular phylogenetic analysis enables us to identify the genomes within a polyploid genome set and to establish the relationships between different genomes.

To determine the position of the species of ×*Agrotrigia* on the tree, we carried out a molecular phylogenetic analysis of these species and probable related taxa, including *Agropyron*, *Elytrigia*, *Elymus*, *Leymus* Hochst., ×*Leymotrigia* Tzvelev, etc. For analysis, we used the nuclear ITS and ETS regions, as well as the chloroplast sequences *trnK–rps16*, *ndhF, trnL–F*, and *psbA–trnH*. Their level of variability is suitable for evolutionary studies at the species level [20,21]. The chloroplast sequences *trnK–rps16*, *ndhF*, *trnL–F*, and *psbA–trnH*, mostly inherited uniparentally and maternally [8,22], are more conserved and evolve more slowly than ITS, but they are important for constructing an independent phylogenetic picture, especially when cases of introgressive hybridization can be assumed. Therefore, it is justified to use these sequences for the phylogenetic reconstruction of species of the tribe Hordeeae, in which hybridization is widespread and is the main evolutionary pathway [9,16,23].

We performed next-generation sequencing (NGS) to study the hybridization pattern in more detail. Modern NGS technologies make it possible to obtain the entire pool of existing marker sequences, including hidden ones left over from ancient hybridization processes and otherwise undetectable. The intragenomic polymorphism of various sequences has been repeatedly and successfully studied in various taxa, including angiosperms (*Hordeum*, *Nepenthes* L.) and gymnosperms (*Abies* Mill.) [24,25,26].

The objective of this study was to evaluate the phylogenetic relationships of the intergeneric hybrid ×*Agrotrigia* (Poaceae) species with the taxa of the parental genera *Agropyron* and *Elytrigia* and other taxa of the tribe Hordeeae, as well as to assess the distribution and conservation of parental genomes in the genomes of hybrid species. We studied three species from herbaria and one putative new species of the intergeneric hybrid ×*Agrotrigia* that we collected.

## 2. Results

### 2.1. Sanger Sequencing Data

The samples analyzed by the Sanger and NGS methods are presented in Table 1.

**Figure 1:**
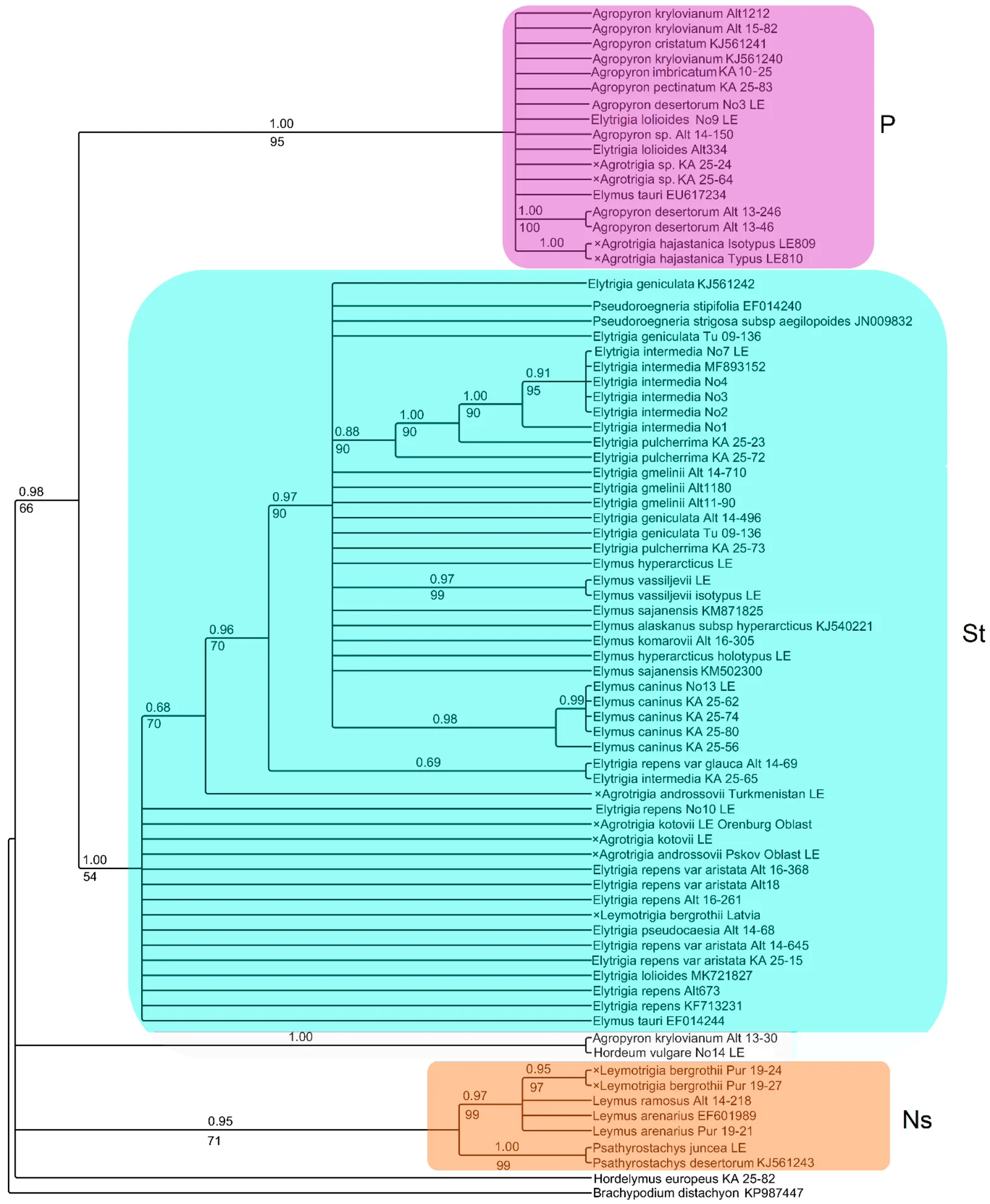
Phylogenetic tree of ×*Agrotrigia* and allied taxa based on ITS1–5.8S rDNA–ITS2 sequences. The number above the branch indicates posterior probability in Bayesian inference, and the number below is the bootstrap index (maximum likelihood method).

The ITS1–5.8S rDNA–ITS2 region contains 607 aligned positions. On the phylogenetic tree constructed from these sequences, the species of the genus ×*Agrotrigia* fall into one large clade divided into two subclades according to their cytogenetically determined genomes most represented in their rDNA (Figure 1). The samples of ×*Agrotrigia hajastanica* (Tzvelev) Tzvelev and ×*Agrotrigia* sp. from Stavropol Krai belong to a larger subclade that includes species of the genus *Agropyron* (P genome) (PP = 1, BS = 95). The sequences of *Elymus tauri* (Boiss. & Balansa) Melderis and *Elytrigia lolioides* (Kar. & Kir.) Nevski also belong to this clade, which indicates that these species also have previously unrecognized P genomes. ×*Agrotrigia androssovii* (Roshev.) Tzvelev from Pskov Oblast (Russia) and ×*A. kotovii* Tzvelev are part of the other subclade (St genome), which unites all other species of *Elymus* L. s. l. and *Elytrigia* s. l. (PP = 1, BS = 54) included in the analysis, *Pseudoroegneria*, and ×*Leymotrigia bergrothii* (H.Lindb.) Tzvelev from Latvia. However, the resolution within this clade is rather poor.

×*Leymotrigia bergrothii* samples from the Arkhangelsk region are grouped with *Leymus* species (Ns genome, PP = 0.97, BS = 99) and *Psathyrostachys* Nevski species, and they form a separate clade (PP = 1, BS = 99) (Figure 1).

**Figure 2:**
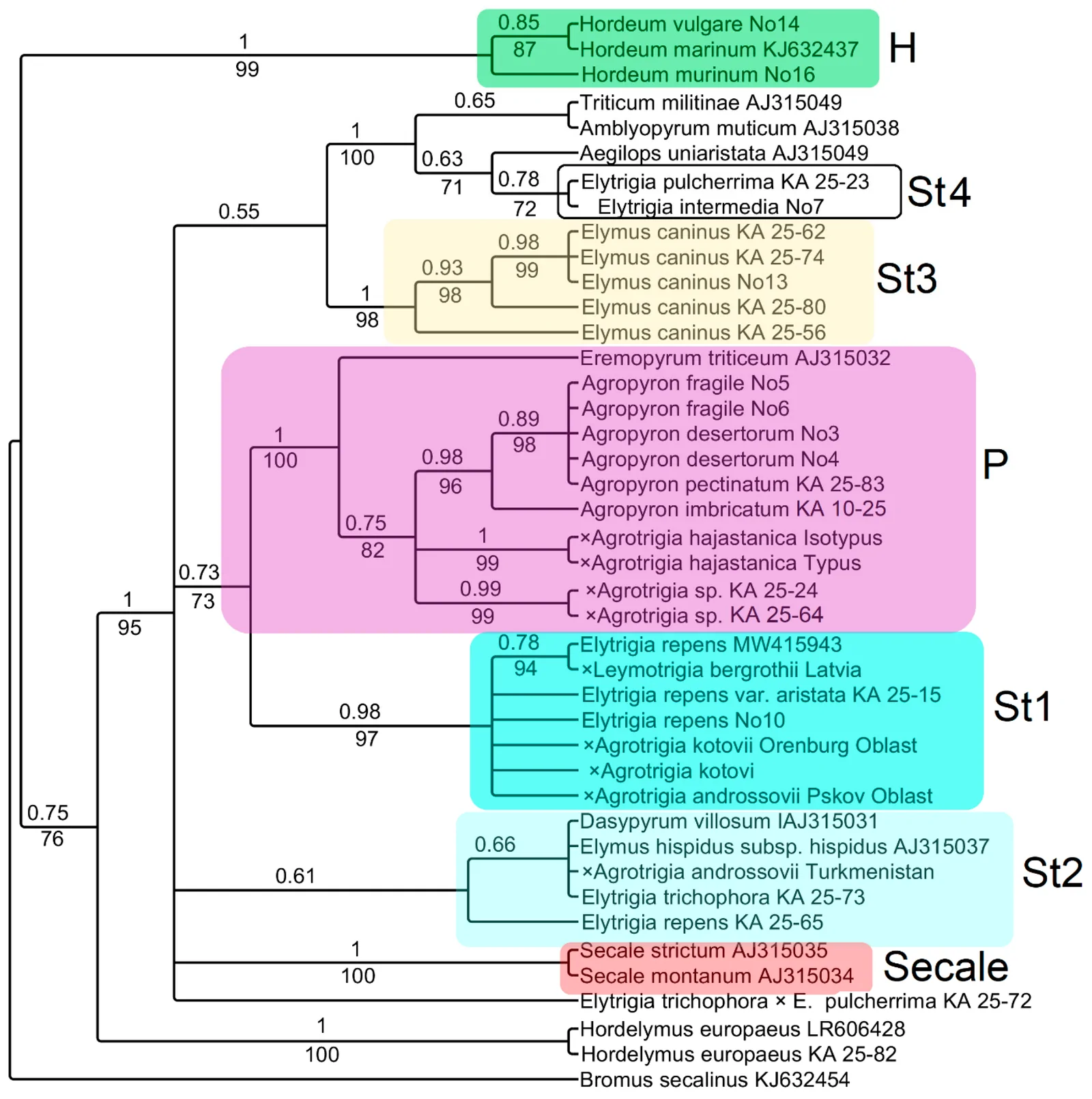
Phylogenetic tree of ×*Agrotrigia* and allied taxa based on ETS sequences. The number above the branch indicates posterior probability in Bayesian inference, and the number below is the bootstrap index (maximum likelihood method).

The ETS sequences comprise 828 aligned positions. The ETS sequences of the *Agropyron* species corresponding to the P genome and of *Elytrigia repens* with the St genome variant (St1) form a single, moderately supported clade (PP = 0.73, BS = 73) (Figure 2). Both samples of *Agrotrigia* sp. from Stavropol Krai and ×*A. hajastanica* each form a separate subclade (PP = 1, BS = 0.99; PP = 0.99, BS = 99, respectively) within the larger subclade, together with *Agropyron* and *Eremopyrum* Jaub. & Spach (PP = 1, BS = 100). ×*Agrotrigia kotovii* and ×*A. androssovii* from Pskov Oblast are grouped with *Elytrigia repens*, as on the ITS tree, and with the ×*Leymotrigia bergrothii* sample from Latvia, forming the subclade corresponding to the St1 genome (PP = 0.98, BS = 97). The sample of ×*A. androssovii* from Turkmenistan falls into the clade with *Dasypyrum villosum* (L.) P.Candargy, *Elymus hispidus* (Opiz) Melderis subsp. *hispidus*, *Elytrigia trichophora* (Link) Nevski, and *E. repens* from the Stavropol region (PP = 0.61) (St2). At the same time, sequences of *Elymus caninus* L. form a separate subclade (PP = 1, BS = 100; PP = 1, BS = 98, respectively) within one weakly supported clade (PP = 0.55), distant from *Elytrigia repens* (St3). The subclade containing *Elytrigia pulcherrima* also includes *Triticum militinae* Zhuk. & Migush. and *Aegilops uniaristata* Vis. from the GenBank database (St4).

The *trnL–trnF* region consists of the *trnL* gene intron and the *trnL–trnF* intergenic spacer, comprising 1159 aligned positions.

**Figure 3:**
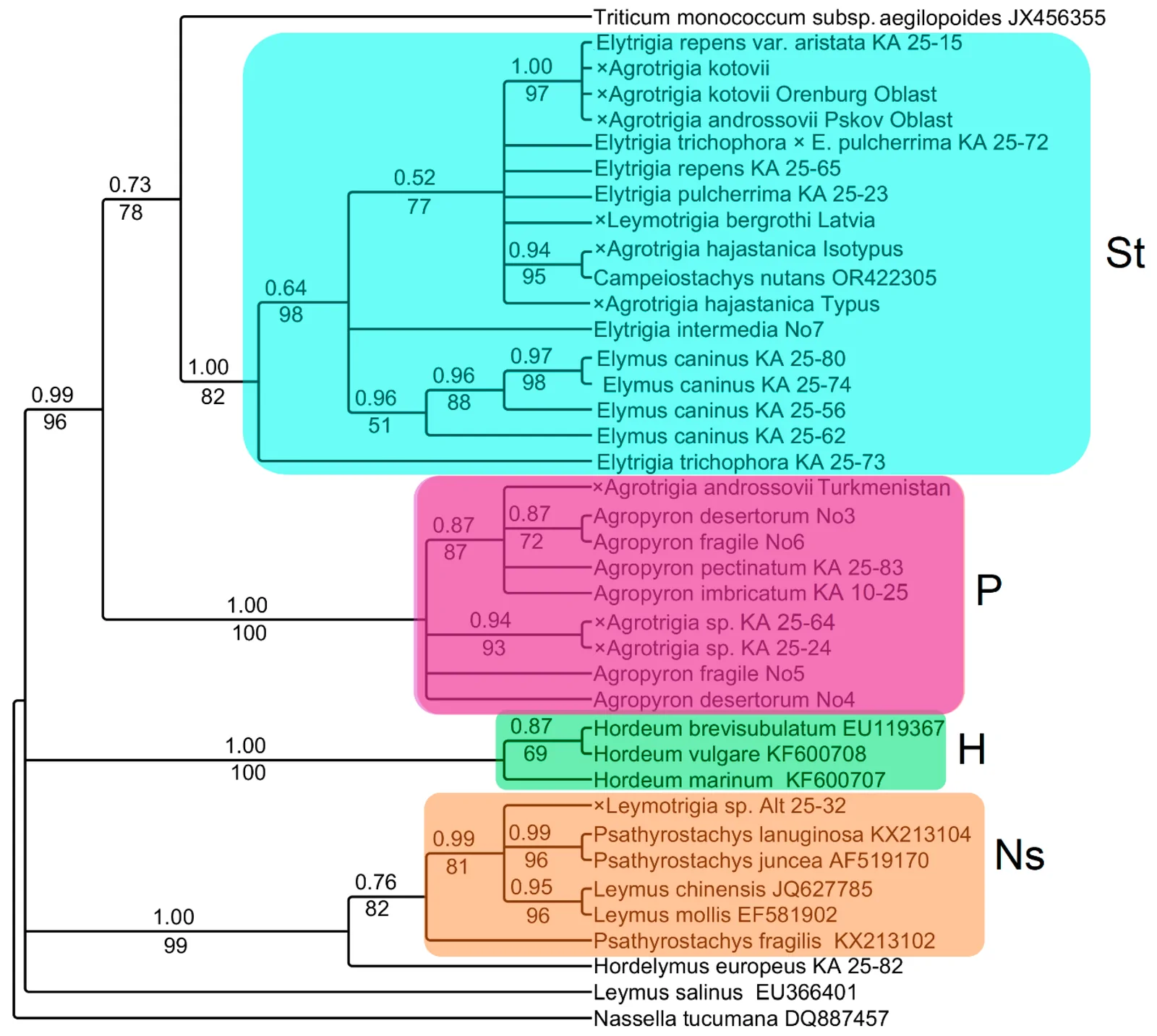
Phylogenetic tree of ×*Agrotrigia* and allied taxa based on *trnL–trnF* sequences. The number above the branch indicates posterior probability in Bayesian inference, and the number below is the bootstrap index (maximum likelihood method).

The ×*Agrotrigia* sp. sample from Stavropol Krai, along with the ×*A. androssovii* sample from Turkmenistan, falls into the clade with the *Agropyron* samples, therefore representing the P genome (PP = 1, BS = 100) (Figure 3). All other samples of the genus ×*Agrotrigia* fall into the other clade, where both samples of ×*A. kotovii* and ×*A. androssovii* from Pskov Oblast are grouped with the species of the genera *Elytrigia* and *Elymus* possessing the St genome (PP = 1, BS = 82). These samples form a monophyletic group with *Elytrigia repens* var. *aristata* (PP = 1, BS = 97), while one of the samples of ×*A. hajastanica* groups with *Campeiostachys nutans* (Griseb.) B. R. Baum, J. L. Yang & C. Yen from the GenBank database (PP = 0.94, BS = 95). *Elytrigia repens* within the large clade of St genomes diverges separately from *E. intermedia* and *Elymus caninus* (Figure 3).

**Figure 4:**
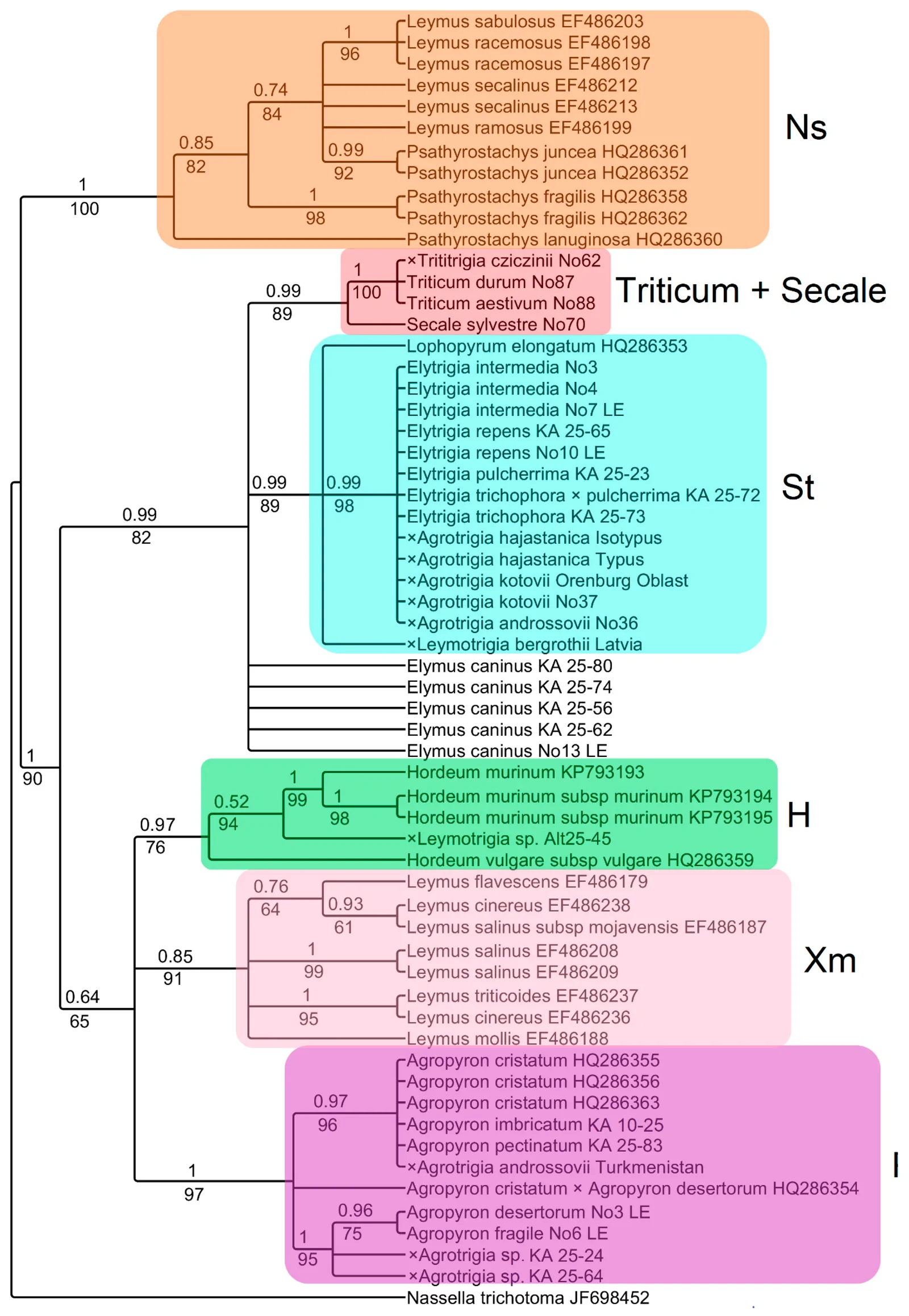
Phylogenetic tree of ×*Agrotrigia* and allied taxa based on *trnK*–*rps16* sequences. The number above the branch indicates posterior probability in Bayesian inference, and the number below is the bootstrap index (maximum likelihood method).

The *trn*K–*rps*16 region consists of 629 aligned positions. The samples of ×*Agrotrigia* sp. from Stavropol Krai enter a highly supported subclade (PP = 0.95, BS = 100) with *Agropyron desertorum* and *A. fragile* samples within a clade corresponding to the P genome (PP = 1.0, BS = 97) (Figure 4). ×*Agrotrigia androssovii* from Turkmenistan, by contrast, groups with *Agropyron cristatum*, *A. imbricatum* Roem. & Schult., and *A. pectinatum* P.Beauv. (PP = 0.97, BS = 96). Both samples of ×*Agrotrigia hajastanica*, and ×*A. kotovii*, as well as ×*A. androssovii* from Pskov Oblast, are found in a common subclade with *Elytrigia repens*, *E. intermedia*, and *E. pulcherrima* (PP = 0.99, BS = 98), which indicates a common maternal origin from the St genome bearer. The species of the genus *Agropyron* (bearers of the P genome) are included in a common weakly supported clade with the species of the genus *Hordeum* (H genome) and some species of the genus *Leymus* (Xm genome) and are distant from the species of the genus *Psathyrostachys* (Ns genome).

**Figure 5:**
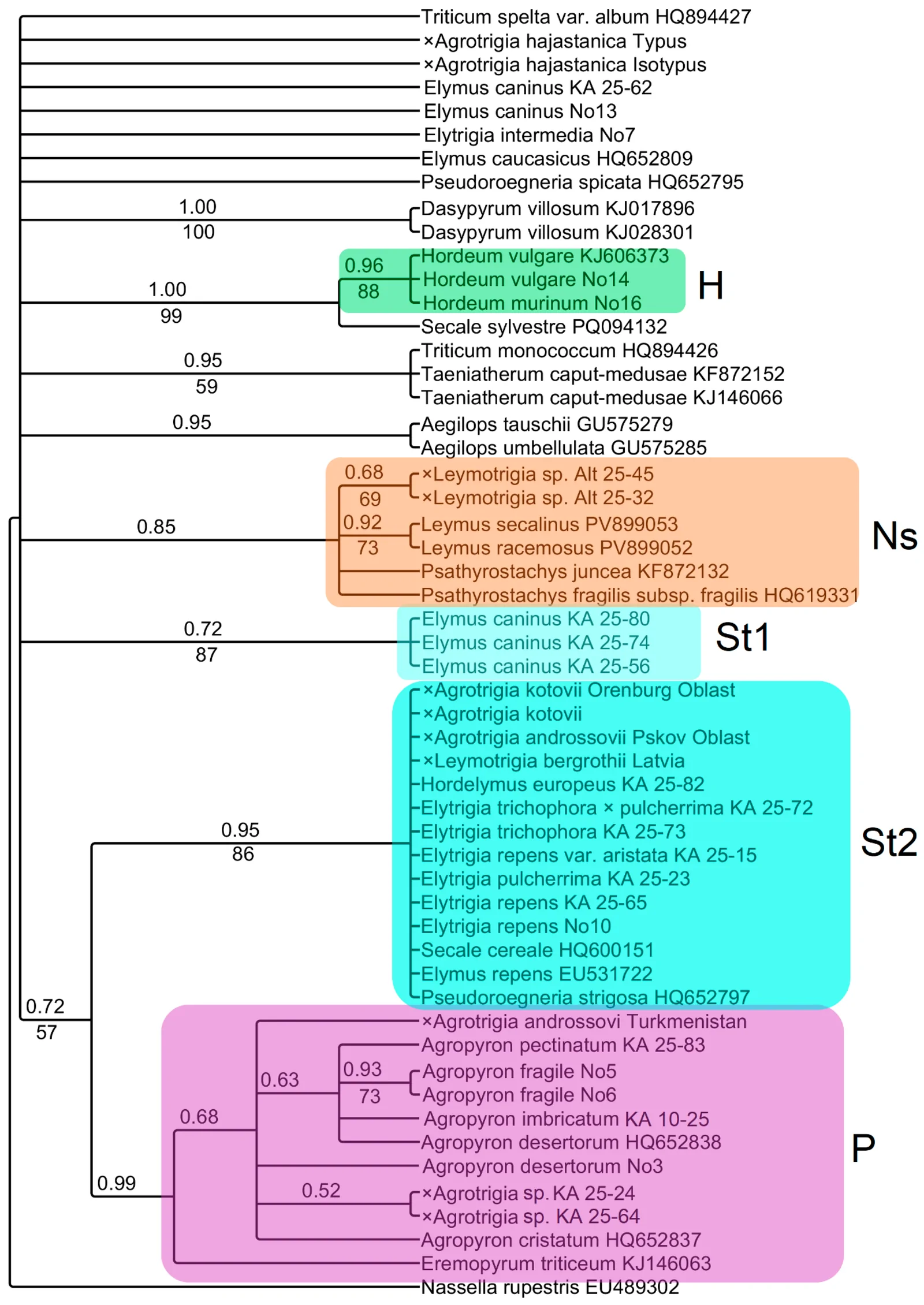
Phylogenetic tree of ×*Agrotrigia* and allied taxa based on *trnH*–*psbA* sequences. The number above the branch indicates posterior probability in Bayesian inference, and the number below is the bootstrap index (maximum likelihood method).

The *trnH–psbA* sequences contain 705 aligned positions. The species of the genus ×*Agrotrigia* on the corresponding tree are also divided into clades according to their maternal genomes (Figure 5). Thus, ×*Agrotrigia androssovii* from Turkmenistan and ×*Agrotrigia* sp. from Stavropol Krai group with the species of the genus *Agropyron* and *Eremopyrum triticeum* (Gaertn.) Nevski (P genome) (PP = 0.99), whereas ×*A. kotovii* and ×*A. androssovii* from Pskov Oblast form a common clade with the species of the genus *Elytrigia* (St genome) (PP = 0.95, BS = 86).

**Figure 6:**
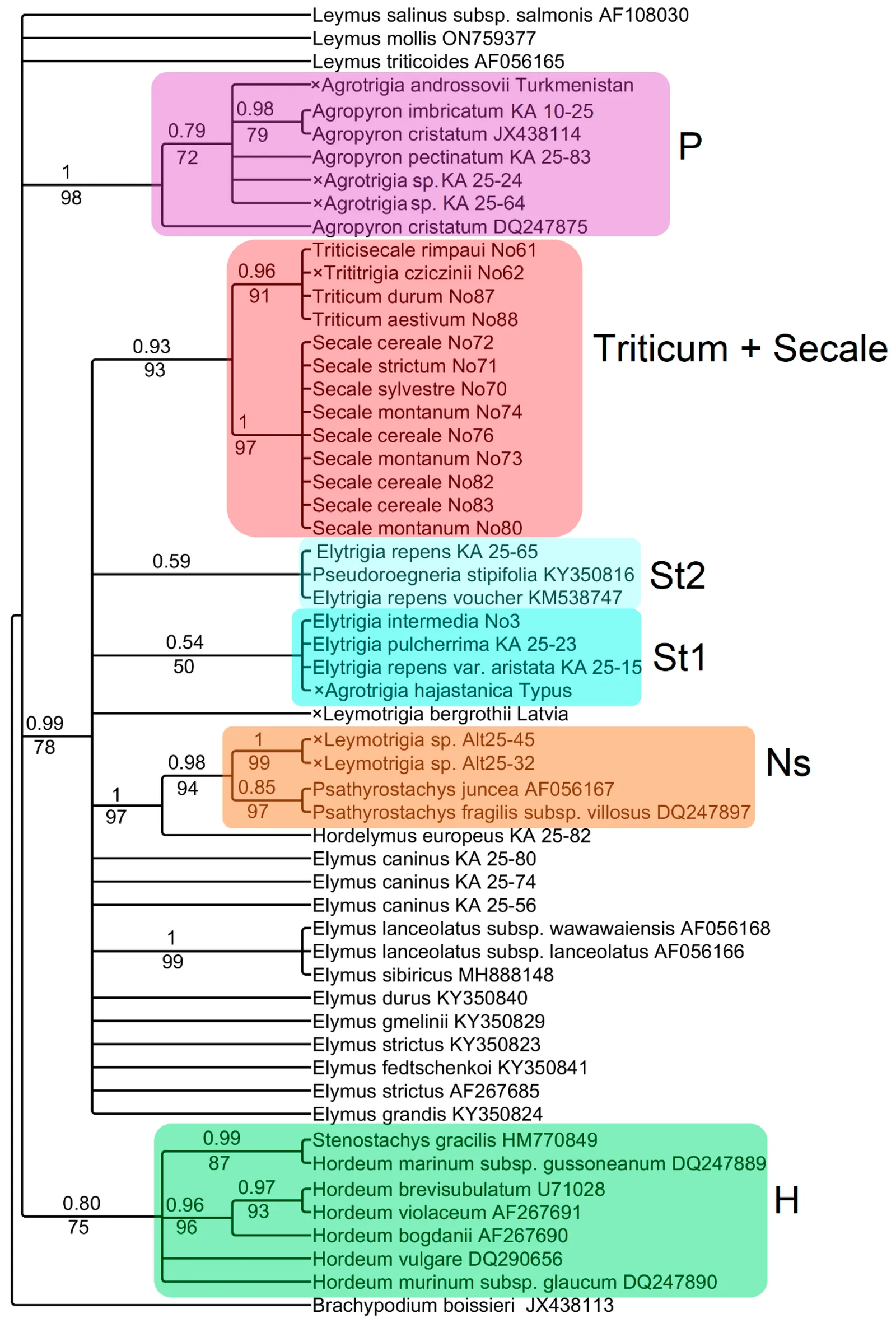
Phylogenetic tree of ×*Agrotrigia* and allied taxa based on *ndhF* sequences. The number above the branch indicates posterior probability in Bayesian inference, and the number below is the bootstrap index (maximum likelihood method).

The *ndhF* sequences are more conserved than other chloroplast markers used in our analyses. They comprise 670 aligned positions. ×*Agrotrigia androssovii* from Turkmenistan and ×*Agrotrigia* sp. from Stavropol Krai are close to the species of the genus *Agropyron* (PP = 1, BS = 98), while ×*A. hajastanica* groups with *Elytrigia repens* var. *aristata*, *E. intermedia*, and *E. pulcherrima* × *E. trichophora* (PP = 0.54, BS = 50) (Figure 6). However, *ndhF* sequences of *Elytrigia repens* var. *aristata* from Stavropol Krai and from the GenBank database diverge separately (PP = 0.59).

### 2.2. NGS Data

We constructed a ribotype network using the statistical parsimony algorithm in TCS 1.2.1 [27] and visualized it in TCS BU [28]. Major ribotypes are presented in Table 2. Sequences of the major ribotypes (more than 1000 reads) are presented in Table 3.

**Figure 7:**
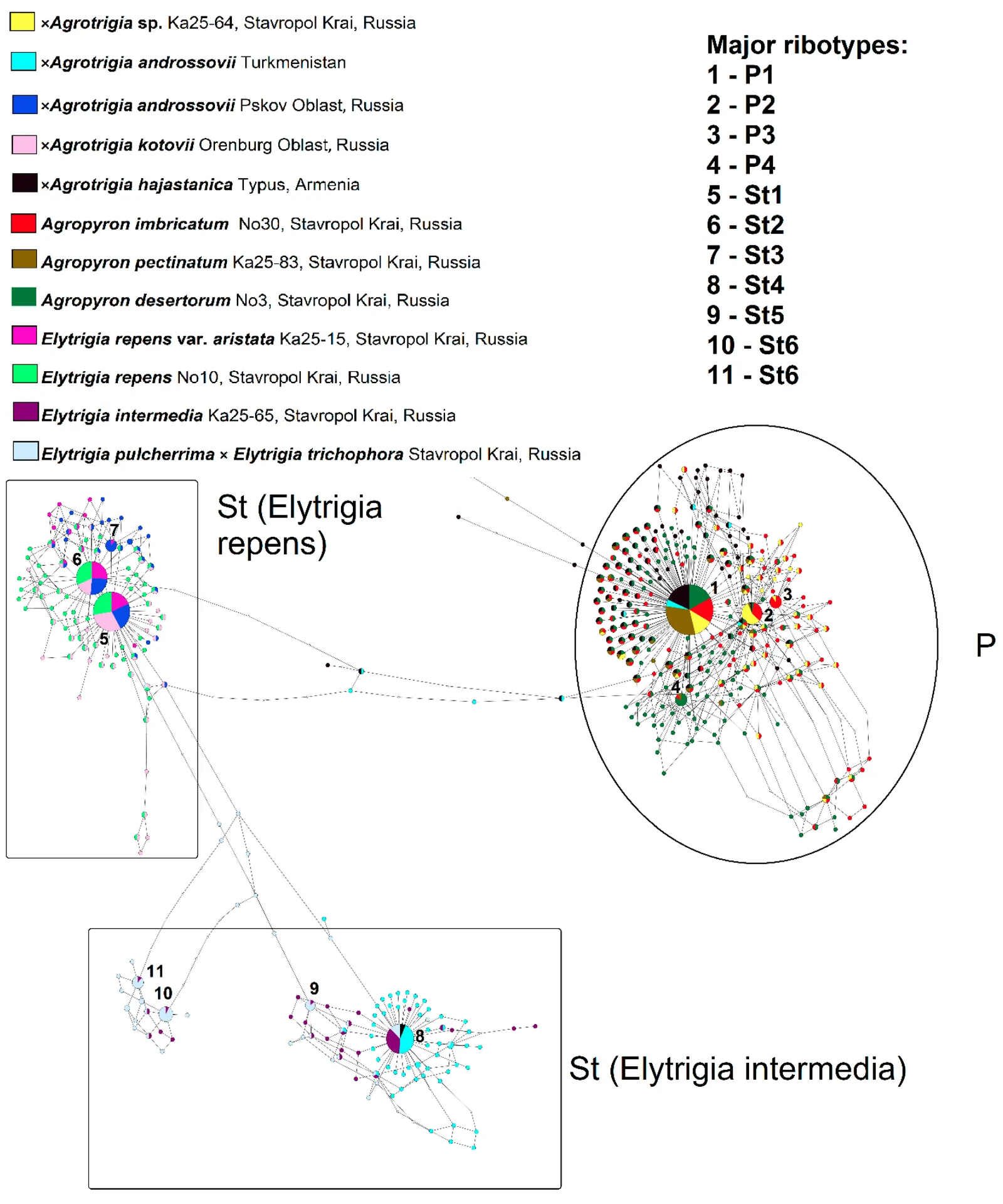
Ribotype network of the ×*Agrotrigia* species and its putative parents based on NGS data (18S–ITS1–5.8S rDNA sequences). The diameter of the circles is proportional to the percentage of the major ribotype to the total number of reads in the sample. The dots on the lines connecting the ribotypes indicate by how many steps (single nucleotide substitutions (SNPs) and/or indels) the ribotypes differ.

The resulting network reflects the relationships of ribotypes in the rDNA of species of the hybrid genus ×*Agrotrigia* and some related genera (Figure 7). We consider major ribotypes to be those ITS1 sequence variants that are represented by more than 1000 reads per rDNA pool, with the exception of the *Elytrigia trichophora* × *E. pulcherrima* sample, for which we consider ribotypes represented by more than 200 reads. The main ribotype of ×*Agrotrigia* sp. from Stavropol Krai (1786 reads, 21% of the total number of reads within the sample) is identical to the main ribotypes of *Agropyron imbricatum* (6563 reads, 26%), *A. pectinatum* (3888 reads, 51%), *A. desertorum* (7326 reads, 28%), and ×*Agrotrigia hajastanica* (4891 reads, 30%) and the second most frequent ribotype of ×*Agrotrigia androssovii* from Turkmenistan (1004 reads, 7%). We call this ribotype P1 (Figure 7). The ribotype P2, most represented in *Agropyron* species, is common as the second major ribotype for *Agropyron imbricatum* (2574 reads, 10%), as well as for ×*Agrotrigia* sp. from Stavropol Krai (1404 reads, 17%) and for *Agropyron desertorum*, *A. pectinatum*, and ×*Agrotrigia hajastanica* as minor fractions. The third major ribotype (P3) is identical to the third ribotype of *Agropyron imbricatum* (2220 reads, 8%) and the minor ribotype of ×*Agrotrigia* sp. from Stavropol Krai. Ribotype P4 is shared with *Agropyron desertorum* (the second most represented ribotype, 1970 reads, 7%) and with *A. imbricatum* and *A. pectinatum* (minor fractions of the ribotypes).

The main ribotype of ×*Agrotrigia androssovii* from Pskov Oblast (2707 reads, 23%) is identical to the main ribotypes of ×*A. kotovii* (3449 reads, 30%) and *Elytrigia repens* from the Stavropol Krai (3437 reads, 27%) and the second most represented ribotype of *E. repens* var. *aristata* (1088 reads, 18%). This ribotype belongs to the *Elytrigia* rDNA pool and can be called St1. The second most represented ribotype of ×*Agrotrigia androssovii* from Pskov Oblast (2126 reads, 18%) is identical to the second major ribotype of *Elytrigia repens* from Stavropol Krai (2762 reads, 22%), the second major ribotype of ×*A. kotovii* (1376 reads, 12%) and the main ribotype of *Elytrigia repens* var. *aristata* (1113 reads, 18%). It can be called St2. The third major ribotype of ×*Agrotrigia androssovii* from Pskov Oblast (1461 reads, 8% of the total number of reads), designated St3, is common with *Elytrigia repens* var. *aristata* from Stavropol Krai (as a minor ribotype).

The main ribotype of ×*Agrotrigia androssovii* from Turkmenistan (3679 reads, 27%) belongs to the pool of *Elytrigia intermedia* (=*Thinopyrum intermedium*) ribotypes. It is identical to the main ribotype of *Elytrigia intermedia* from Stavropol Krai (1096 reads, 20%) and the third major ribotype of *Elytrigia pulcherrima* × *E. trichophora* (333 reads, 7%), as well as to the minor ribotype of ×*Agrotrigia hajastanica*. The fourth major ribotype of *E. pulcherrima* × *E. trichophora* (276 reads, 6%) also belongs to the pool of *E. intermedia* ribotypes and is identical to the minor ribotype of *E. intermedia*. The main ribotype of the *E. pulcherrima* × *E. trichophora* hybrid (618 reads, 13%) is identical to the minor ribotype of *E. intermedia* and forms its own ribotype subnetwork together with some *E. intermedia* ribotypes. The second major ribotype of *E. pulcherrima* × *E. trichophora* (348 reads, 8%), identical to the minor ribotype of *E. intermedia*, also belongs to this subnetwork.

**Figure 8:**
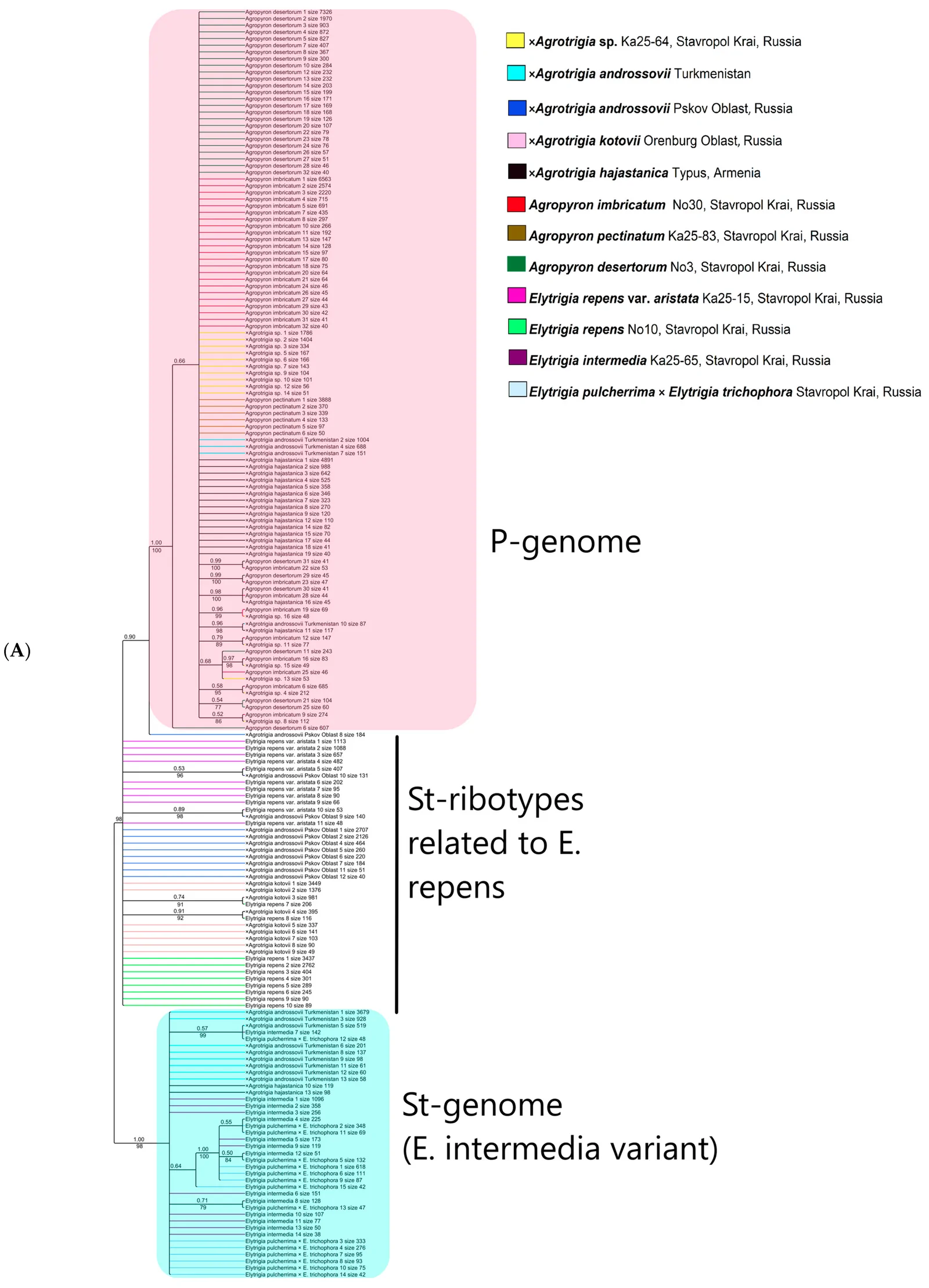

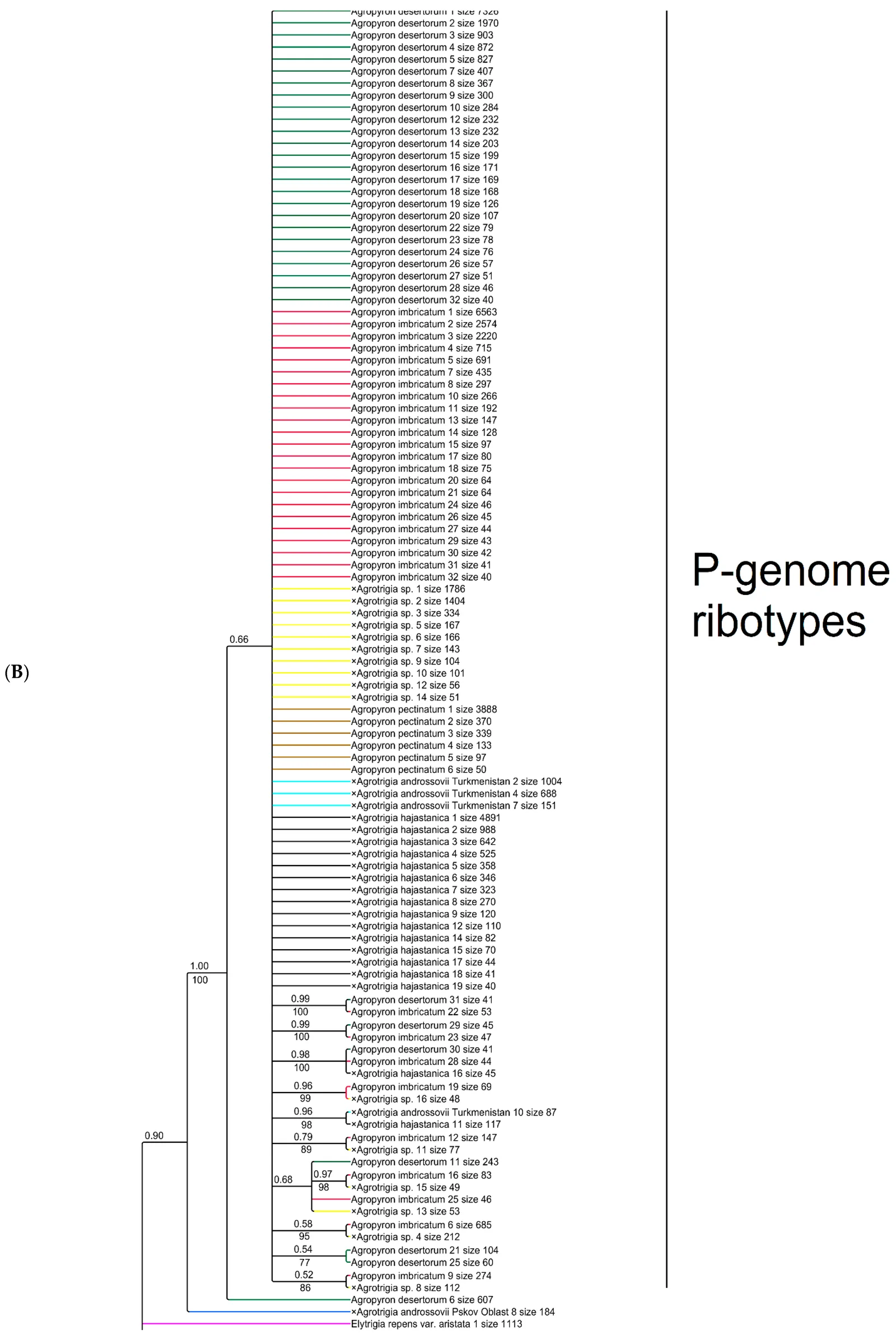

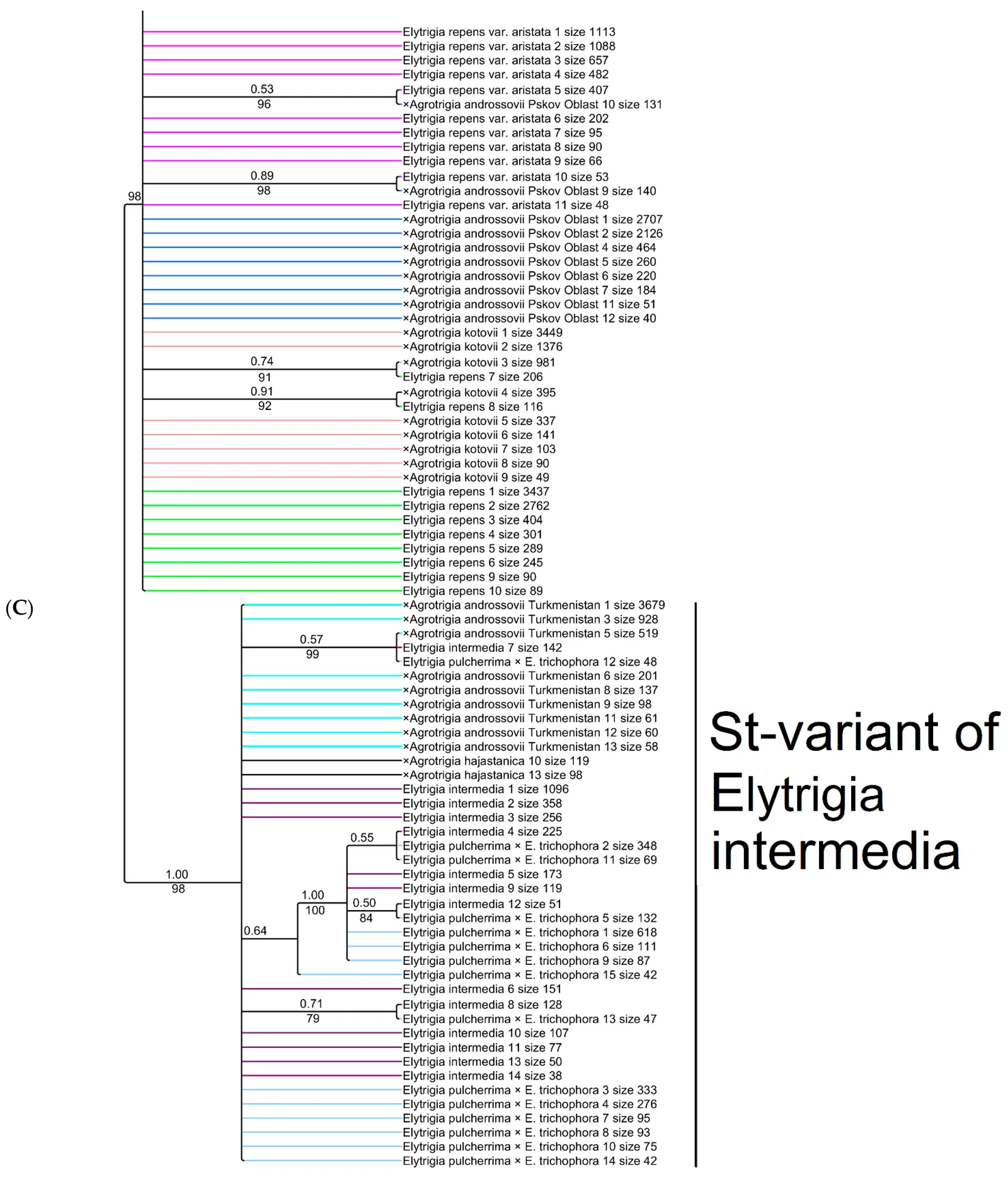
(**A**) Phylogenetic tree of the ribotypes showing the relationship between ×*Agrotrigia* and allied species (threshold is 40 reads per rDNA pool). Index above the branch indicates posterior probability in Bayesian inference, and the number below is the bootstrap index (maximum likelihood method). (**B**) P genome-related ribotypes. (**C**) St genome-related ribotypes. Size in the ribotype name shows the number of reads per rDNA pool.

The ribotype tree constructed with a threshold of up to 40 reads per rDNA pool separates large clades corresponding to the genomes of polyploid species (P, St and others) (Figure 8A). A large clade is characterized by the P genome, which is present in species of the genus *Agropyron* and in hybrids containing this genome (PP = 1, BS = 100) (Figure 8B). In addition to ribotypes of *Agropyron*, this clade also includes those of the ×*Agrotrigia* sp. sample from Stavropol Krai, ×*Agrotrigia hajastanica*, and ×*Agrotrigia androssovii* from Turkmenistan. One minor ribotype of ×*Agrotrigia androssovii* from Pskov Oblast is sister to the large *Agropyron* clade, which diverges only in Bayesian analysis (PP = 0.9) (Figure 8B). Sequences characteristic of the St genome variant that comprises *Elytrigia repens* and its hybrids occupy an uncertain position on the tree (Figure 8C). Some minor ribotypes of ×*Agrotrigia androssovii* from Pskov Oblast form small subclades with ribotypes of *Elytrigia repens* var. *aristata* (PP = 0.53, BS = 96; PP = 0.89, BS = 98) (Figure 8C). At the same time, minor ribotypes of *Elytrigia repens* s. str. group with those of ×*Agrotrigia kotovii* (PP = 0.74, BS = 91; PP = 0.91, BS = 92). Ribotype variants of *Elytrigia intermedia* and its hybrids form a separate clade (PP = 1, BS = 98) (Figure 8C). It includes the ribotypes of ×*Agrotrigia androssovii* from Turkmenistan, *Elytrigia intermedi*a, and *E. pulcherrima* × *E. trichophora*. Some minor ribotypes of these species form a common subclade (PP = 0.57, BS = 99) (Figure 8C).

**Figure 9:**
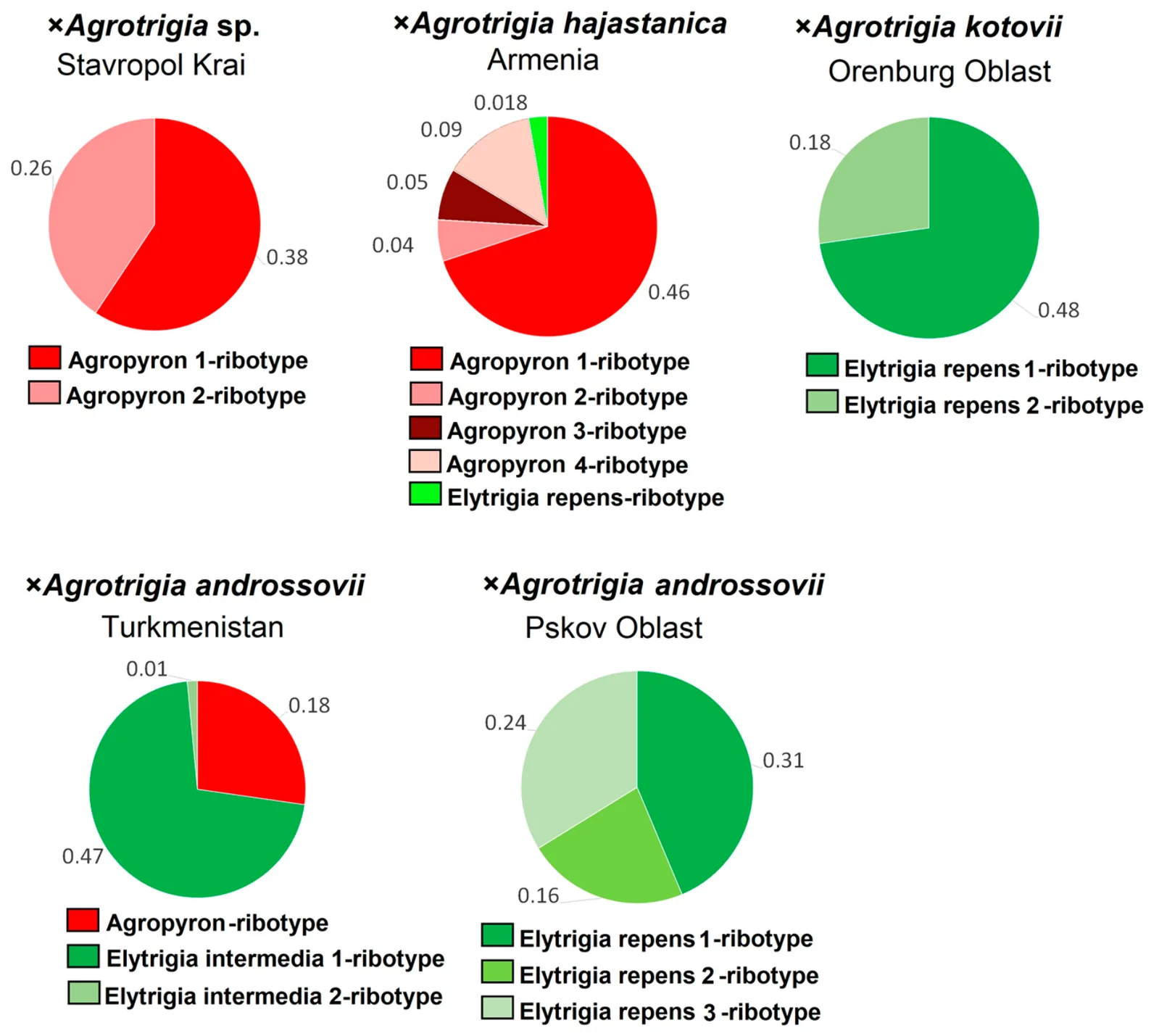
Ribotype composition of the studied ×*Agrotrigia* species (threshold is 10 reads per rDNA pool). The percentage is calculated relative to the total number of rDNA reads, so the sum will be less than 100%.

**Figure 10:**
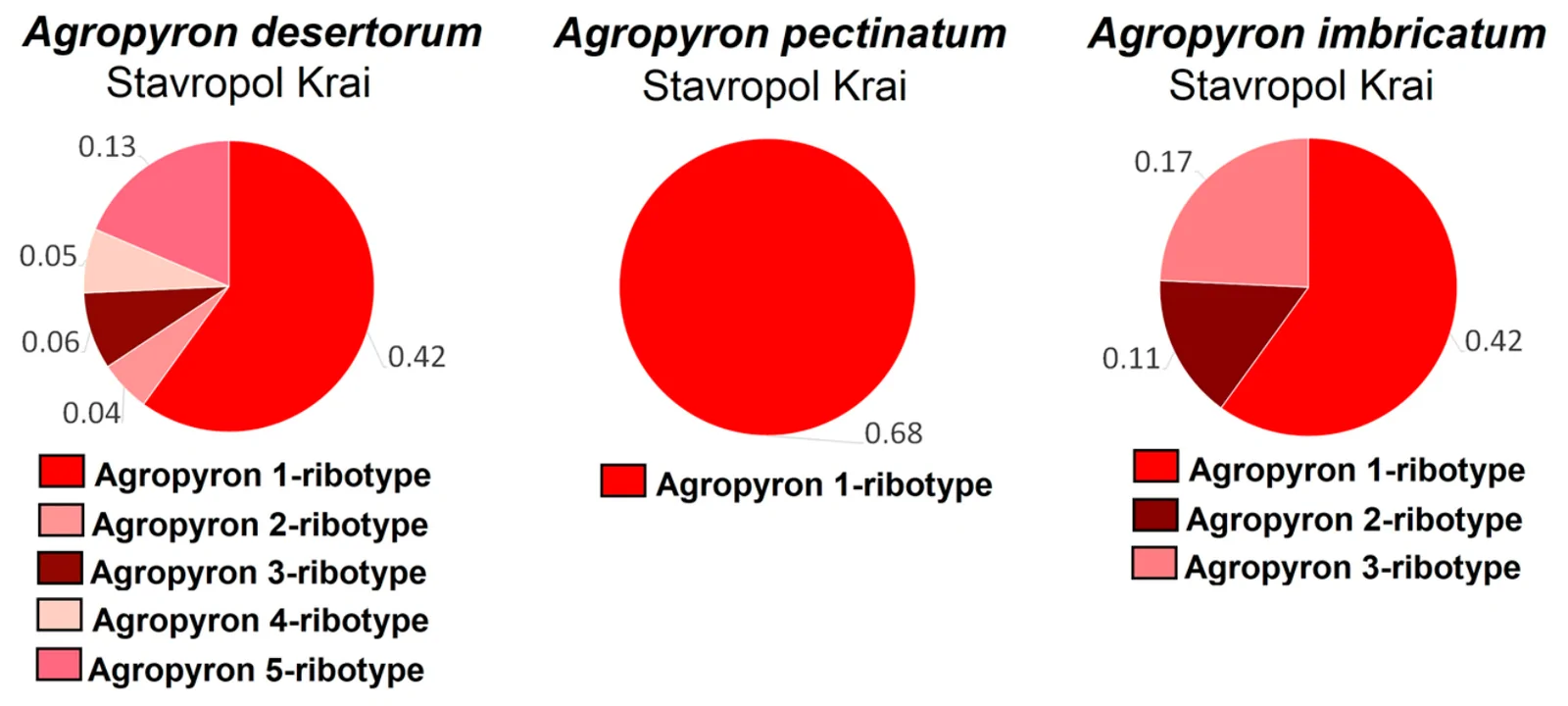
Ribotype composition of the studied *Agropyron* species (threshold is 10 reads per rDNA pool). The percentage is calculated relative to the total number of rDNA reads, so the sum will be less than 100%.

**Figure 11:**
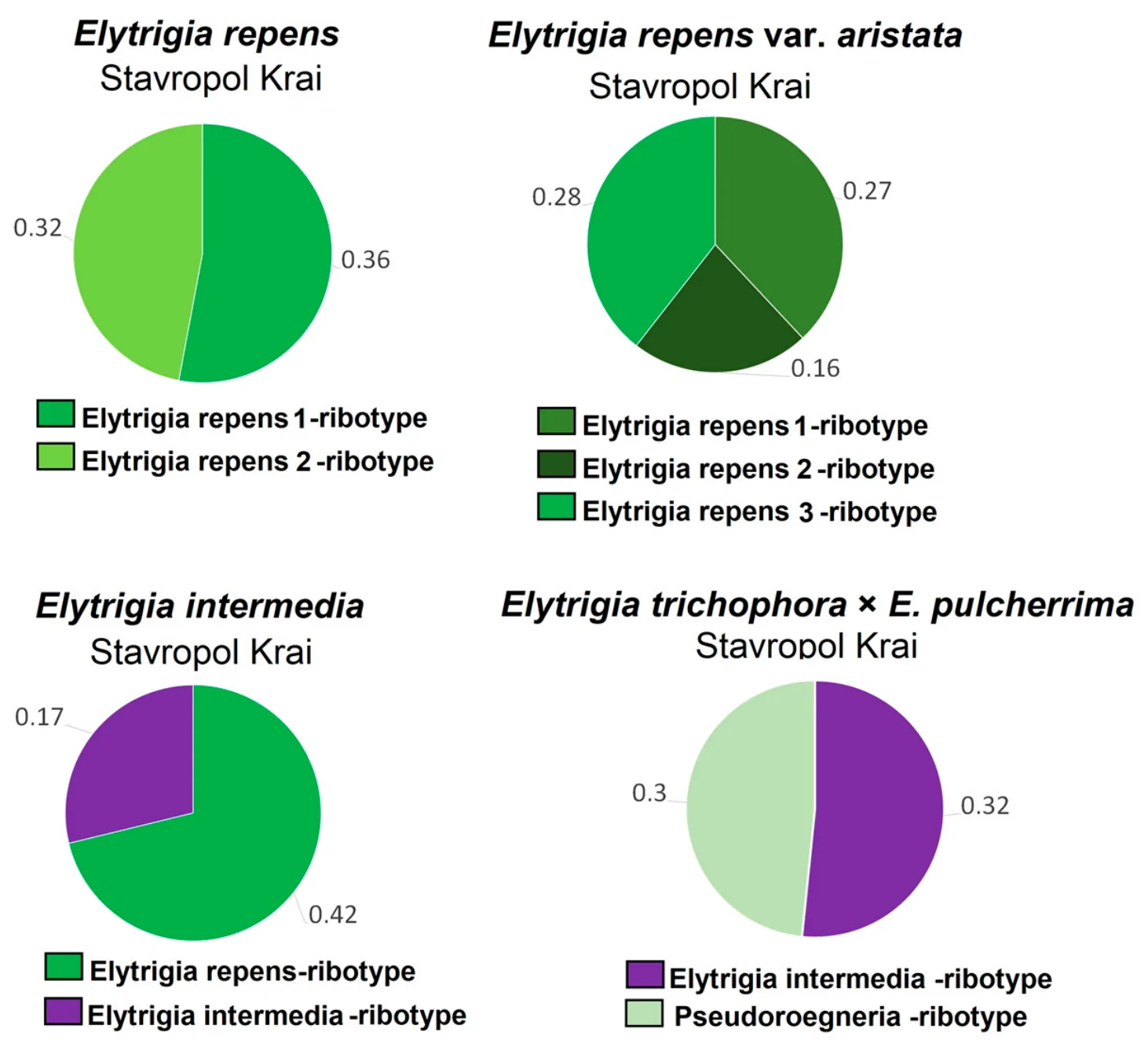
Ribotype composition of the studied *Elytrigia* species (threshold is 10 reads per rDNA pool). The percentage is calculated relative to the total number of rDNA reads, so the sum will be less than 100%.

We also examined the composition of ribotypes of each sample included in the NGS analysis (Figure 9, Figure 10 and Figure 11). The composition of ribotypes reflects the proportion of each ribotype relative to the total number of reads in the sample in the largest clusters calculated in Arlequin 3.5.1.2 [29] using the p-distance matrix and the minimum spanning tree method. The ribotypes in these calculated clusters were named according to the most similar sequence from the GenBank database (https://www.ncbi.nlm.nih.gov/nuccore/?term=) (accessed on 1 September 2025).

The sample of ×*Agrotrigia* sp. from Stavropol Krai, according to its rDNA, consists of *Agropyron*-type ribotypes (P genome) only, within which two clusters are distinguished: *Agropyron*-1 and *Agropyron*-2 ribotypes (38% and 26% of the total number of reads, respectively; Figure 9). ×*Agrotrigia androssovii* (Turkmenistan) has ribotypes of both the *Elytrigia intermedia* type (47% and 1%) and *Agropyron* type (18%). ×*Agrotrigia androssovii* from Pskov Oblast, on the contrary, consists only of *Elytrigia*-type ribotypes, belonging to the *Elytrigia repens* group, which are divided into three clusters (31%, 16%, and 24%). ×*Agrotrigia kotovii* also bears rDNA of the *Elytrigia repens* type only (48% and 18%). The sample of ×*Agrotrigia hajastanica* carries four clusters of *Agropyron*-type ribotypes (46%, 4%, 5%, 9%) and one cluster of the *Elytrigia repens* type (1.8%) (Figure 9).

The composition of ribotypes is rather constant in the putative parental taxa (Figure 10 and Figure 11). The sample of *Agropyron pectinatum* has the rDNA of only one group of *Agropyron* ribotypes (68% of the total number of reads, Figure 10). *Agropyron imbricatum* contains three groups of *Agropyron* ribotypes (42%, 11%, and 17%). *Agropyron desertorum* is the most diverse in terms of the number of ribotype clusters (42%, 4%, 6%, 5%, and 13% of the total number of reads) (Figure 10). *Elytrigia repens* var. *aristata*, in its rDNA, consists only of *Elytrigia repens* ribotypes (27%, 16%, and 28%, Figure 11). *Elytrigia repens* s. str. contains two clusters of *Elytrigia repens* ribotypes (36% and 32%) according to its rDNA. The studied *Elytrigia intermedia* sample unexpectedly turns out to be a hybrid according to rDNA: 42% of its read composition is *Elytrigia repens* ribotypes, and 17% is *E. intermedia* ribotypes (Figure 11). We also included a sample of the hybrid *E. pulcherrima* × *E. trichophora* in the analysis. It contains rDNA of *E. intermedia* (32%) and rDNA related to species of the genus *Pseudoroegneria* (30% of the total number of reads within the sample).

## 3. Discussion

### 3.1. Overview of the Hybrid Genus and Putative Parental Taxa

The genus ×*Agrotrigia* now includes four species: ×*A. androssovii*, ×*A. hajastanica*, ×*A. kotovii*, and ×*A. czernjaew*i (Širj. & Lavrenko) Sutorý (*Elytrigia repens* × *Agropyron dasyanthum* Ledeb.). We were unable to find specimens of the last species. We examined and compared the morphological features of the available specimens of the genus ×*Agrotrigia* using the corresponding literature [30]. ×*Agrotrigia androssovii* is the type of the genus; it is considered a hybrid between *Elytrigia intermedia* and *Agropyron cristatum* s.l. based on morphological characteristics. It was described from Turkmenistan and is also found in northwestern Russia (Pskov Oblast). ×*Agrotrigia androssovii* is distinguished by more widely spaced spikelets, which places it closer to the genus *Elytrigia*. ×*Agrotrigia hajastanica* is a hybrid between *Elytrigia repens* s. l. and *Agropyron cristatum* s. l. It was described from Armenia but has already been recorded in many places in the south of the European part of Russia, usually forming loose tufts, often with short creeping shoots. Its spikes and spikelets have an intermediate structure between those of the parental genera [30]. ×*Agrotrigia hajastanica* is also reported for the Russian Far East [31], from where the chromosome number for ×*A. hajastanica* was first given (2n = 42: Amur Region, [31]).

In ×*A. kotovii*, the parental taxa are *Elytrigia repens* s. l. and *Agropyron cristatum* subsp. *sclerophyllum* Novopokr. This hybrid is significantly more similar in habit to *E. repens*, but its glumes are quite typical of the genus *Agropyron*, often with cilia along the keel [14]; it is found in the southern regions of Russia.

During a summer botanical expedition in 2025 to the foothills of North Caucasus (Russia, Stavropol Krai), we found an unusual specimen of Hordeeae grass: its spike features slightly resembled those of *Psathyrostachys* and *Hordeum*, though it was mesophytic in habit. The spikelets and lemmas were also similar to those of *Agropyron*, especially in their form and pubescence; its spikes were intermediate between those of *Agropyron*, *Hordeum* and *Elytrigia*. Thus, this plant exhibited combined morphological characteristics of the genera *Agropyron* and *Elytrigia*, but its habit differed from that of previously described species of the genus ×*Agrotrigia*. Its spikelets were similar to those of *Agropyron* in terms of their form and pubescence. This unusual form of spikes may have arisen because of complex interactions of genes in a hybrid genome. We considered these specimens to be a probable new hybrid species very distinct from other members of the genus ×*Agrotrigia* and included them in our analyses.

### 3.2. Phylogenetic Picture of the Genus Agrotrigia Based on Nuclear and Chloroplast Sequence Data

Our molecular phylogenetic data show the division of the hybrid ×*Agrotrigia* species according to the contribution of maternal genomes to their genome composition. The trees constructed from chloroplast and nuclear markers reflect hybridization events; therefore, we did not concatenate them. The highest resolution is shown by trees constructed based on nuclear genes—ITS and ETS, and by chloroplast genes—constructed based on *trnK*–*rps16*. Only two species—×*A. hajastanica* and ×*A. androssovii* (specimen from Turkmenistan)—obviously show the presence of both parental genomes in the genomic set. They have ribotypes of *Agropyron* and *Elytrigia* origin, though in different proportions. ×*Agrotrigia hajastanica* has a main ribotype that belongs to the *Agropyron* type, and a minor fraction of *E. intermedia* ribotypes, whereas ×*A. androssovii* (specimen from Turkmenistan) shows the opposite situation: the main ribotype belongs to the *E. intermedia* group, while *Agropyron* ribotypes present in a minor fraction. Additionally, ×*Agrotrigia hajastanica* has *Elytrigia intermedia* as the maternal parent (St genome-related chloroplast sequences are very similar to those of *E. repens*), whereas ×*A. androssovii* from Turkmenistan originated on the maternal side from some *Agropyron* species. The sample of ×*A. androssovii* from Pskov Oblast does not correspond to the typical specimen of ×*A. androssovii* because it has only *E. repens* ribotypes and is not even related to *Elytrigia intermedia*. Thus, it may represent another as-yet-undescribed species and not ×*A. androssovii.* The unusual sample from the Caucasus foothills turned out to belong to the genus ×*Agrotrigia*, with almost all marker sequences (both chloroplast and nuclear) belonging to the P genome (*Agropyron*). This unusual find once again confirms that the Caucasus Mountains are one of the world’s centers of speciation and hybridization [30].

**Figure 12:**
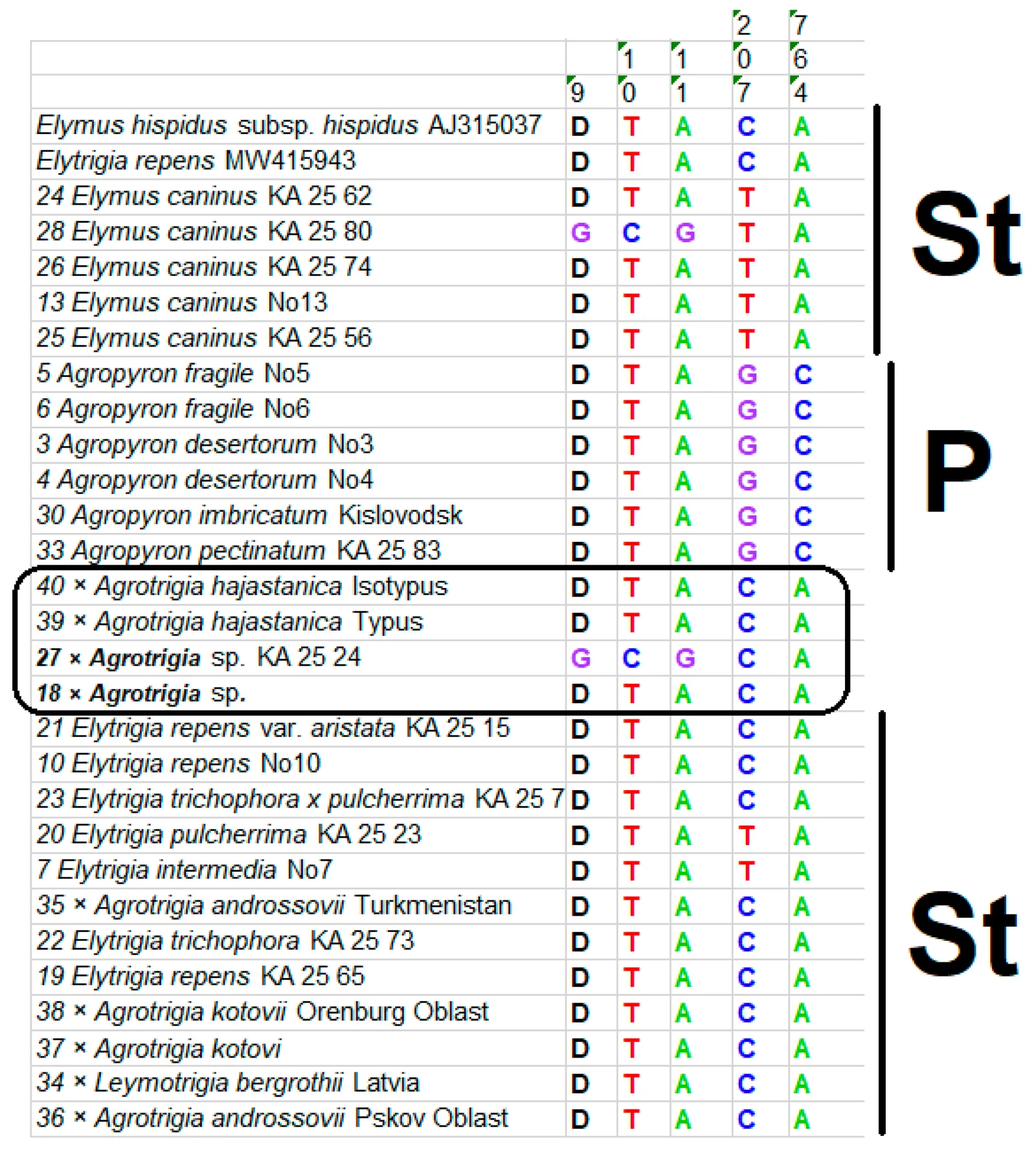
Nucleotide substitutions in ETS sequence separating different genomes. D is a deletion.

In the ETS region, only a few nucleotide substitutions in *Agrotrigia* sp. from Stavropol Krai are common with those of *Elytrigia* and *Elymus*, though most substitutions belong to the *Agropyron* group (Figure 12). This demonstrates a remarkable phenomenon of the almost complete elimination of one of the parental genomes, with probable intragenomic conversion of the remaining genome. The presence of exclusively *Agropyron*-type sequences is not characteristic of other species of ×*Agrotrigia* and further indicates the probable species independence of this sample. The predominance of the sequences originating from the genus *Agropyron* in the genomic set of the sample most likely indicates long-term introgressive hybridization that led to the formation of this specimen and, accordingly, to the stabilization of its polyploid genomic set.

The sample of ×*Agrotrigia kotovii* contains only St genome sequences that are characteristic of *Elytrigia repens*. We did not find any P genome sequences characteristic of *Agropyron cristatum* subsp. *Sclerophyllum*, which was previously considered to be its parental species [30]. This species is likely a fully stabilized hybrid with the complete elimination of one of the parental genomes. In general, stable species formed via more or less distant hybridization often experience sequential elimination of one of the parental genomes, particularly through pseudogenization, which culminates in the complete disappearance of one of the genomes [32,33,34]. In artificial hybrids of *Avena sativa* × *A. macrostachya*, some lines exhibit complete loss of the C genome variant characteristic of the ancient and evolutionarily isolated oat *A. macrostachya*, as we see from cytogenetic data [35]. In our case, the main parental species is undoubtedly *Elytrigia repens*. ×*Agrotrigia kotovii* is likely a “good species” stabilized through repeated introgressive hybridizations.

### 3.3. Notes on Hordeeae Phylogeny and Parental Species of Agrotrigia, Especially About St Genome Bearers

Important data obtained using molecular phylogenetic methods include evidence of the high hybridity of species of the Hordeeae tribe as a whole. The prevailing view in the literature is that genera of the Hordeeae tribe differ in the combinations of genomes inherited from diploid ancestors [15,16,36,37]. However, polyploid representatives of the tribe may actually undergo a series of introgression events, as a result of which, at least according to marker sequence data, the polyploid genomic set may also contain traces of genomes that have not previously been identified in species of certain genera. For example, we observe the presence of P genome sequences characteristic of the genus *Agropyron* in the ITS sequences of *Elytrigia lolioides*, for which the P genome had not previously been demonstrated [18].

Another important and interesting finding is the strong differentiation of the St genomic components in the species of the genera *Elytrigia* s. l. and *Elymus* s. l. Earlier molecular phylogenetic studies have shown that the St genomic component was predominantly amplified in species of these two genera [38,39]. We found a significant difference between the St genome variants, possibly inherited from the diploid *Pseudoroegneria stipifolia* on the one hand and from *Elytrigia repens* (=*Elymus repens*) on the other. Based on ITS sequence analysis, the studied *Elymus* accessions fall into the same clade as the St genome donor, *Pseudoroegneria stipifolia*. At the same time, the ITS sequences of most of the *Elytrigia repens* samples studied by us are more distantly related to those of *P. stipifolia*, falling within a single large clade of *Elymus* + *Elytrigia*. In an earlier paper that examined the origin and genomic composition of the species of the genus *Elytrigia* s. l., a distinction was found between the group including species related to *E. intermedia* (=*Thinopyrum intermedium*) and *Pseudoroegneria spicata* and the group including *P. strigosa*, *P. stipifolia*, and *Elytrigia repens* [18]. However, our data show a somewhat more complex differentiation than that in the work of Yang et al. [18]: *Pseudoroegneria stipifolia* and *Elytrigia intermedia* form a common subclade with *E. gmelinii* and *Elymus caninus*, whereas *Elytrigia repens*, *E. lolioides*, and *Elymus tauri* are quite distant from *P. stipifolia*, occupying an uncertain position on the tree. In addition, the ITSs of *Elytrigia repens* var. *glauca* (the sample from Altai) and *E. intermedia* (the sample from the Caucasus) form a separate subclade. We are likely observing here both a different origin of the St genomic component for different *Elytrigia* affinity groups—partly similar to that described by Yang et al. [18]—as well as evidence of multiple hybridization and introgression. Despite the different origins of *E. repens* and *E. intermedia* [18], they apparently hybridize with each other quite easily, as observed from our ITS sequence analysis. Species from the *E. repens*- and *E. intermedia*-related groups also hybridize well with the species of the genus *Agropyron*, forming a kind of sibling species complex, as only the ×*Agrotrigia androssovii* specimen from Turkmenistan belongs to this species, while the specimen identified as ×*A. androssovii* from the Pskov region likely represents a separate species related to ×*A. kotovii*. However, the maternal St genome of both the *E. intermedia*-related group and the *E. repens*-related group is most likely common.

Our study of the taxa related to ×*Agrotrigia* also showed a different origin of the sample of the hybrid genus ×*Leymotrigia* (previously assigned to ×*Tritordeum* [40]) from the vicinity of Riga, Latvia, compared to that of the sample from the *locus classicus—*the White Sea coast studied by us previously [41]. The ×*Leymotrigia bergrothii* sample from the *locus classicus* was derived from the hybridization of *Elytrigia repens* with *Leymus arenarius*, with its rDNA originating from *L. arenarius* and its chloroplast DNA from *E. repens* [41]. However, specimens of ×*L. bergrothii* from the vicinity of Riga (Latvia) contain only the sequences of *Elytrigia repens* in their rDNA, along with chloroplast sequences of *E. repens*. Thus, when analyzed by the Sanger method, ×*L. bergrothii* from Latvia does not show the presence of different genomes, possibly being a stabilized hybrid. Unusual samples from Altai, possibly belonging to ×*Leymotrigia* sp., show a relationship with *Leymus* (Ns genome), but, unexpectedly, one of the *trn*K–*rps*16 samples turns out to be related to *Hordeum* (noting the previous assignment of ×*Leymotrigia* to ×*Tritordeum* [40]).

### 3.4. Elimination of the Parental rDNA in Different Agrotrigia Species

The varying degree of elimination of parental genomes in the allopolyploid genomic set is associated with the stabilization of the hybrid. In the beginning, newly formed hybrids are in a condition of “genomic shock” characterized by chromosomal rearrangements, changes in the pattern of genome methylation, transcriptome changes, and transposon migration [5,6,34,42,43]. Subsequently, the genome enters a new, stable condition in which the plant can, in turn, enter into new, distant interspecific and intergeneric hybridizations. Among the taxa studied by us, the differential elimination of parental genomes occurred based on the maternal component of the plant genome in all cases except ×*A. androssovii* from Turkmenistan. In this case, the elimination of parental genomes proceeds based on the taxon that could have made the greatest contribution to introgression. As a result, ×*Agrotrigia kotovii* and the specimen of ×*A. androssovii* from Pskov Oblast, which contain, in their rDNA set, *Elytrigia repens* rather than *E. intermedia*, do not contain *Agropyron* sequences. ×*Agrotrigia* sp. from Stavropol Krai may have gone through the stages of intragenomic conversion, retaining only individual substitutions in ETS that are characteristic of *Elytrigia*. Morphologically, it is quite similar to the species of the genus *Agropyron* but differs in the shape of the spike. In addition, we observed that ×*Agrotrigia hajastanica*, like ×*Agrotrigia androssovii* from Turkmenistan, has *Elytrigia intermedia* sequences in its rDNA and not *E. repens*, as was assumed from morphological data. However, the proportions of ribotypes in the rDNA of ×*A. hajastanica* are different: the main ribotype is that inherited from *Agropyron*, rather than that from *Elytrigia*, and, morphologically, ×*A. hajastanica* is closer to *Agropyron* species. In this case, complete intragenomic conversion has not yet occurred; these hybrids are in the process of stabilization.

## 4. Materials and Methods

### 4.1. Plant Materials

The herbarium specimens were collected by the authors during a 2025 summer expedition to Stavropol Krai, Russia. Some specimens were obtained from the LE herbarium collections. Our study included species of the genus ×*Agrotrigia* and their parent taxa, *Agropyron* and *Elytrigia*, as well as related taxa from the tribe Hordeeae—*Elymus*, *Leymus*, *Psathyrostachys*, and others. Some marker sequences for genera of the tribe Hordeeae were obtained from the GenBank database (https://www.ncbi.nlm.nih.gov/nuccore/?term=, accessed on 15 November 2025). A summary of the species included in the analysis is presented in Table 1.

### 4.2. DNA Extraction, Amplification, and Sequencing (Sanger Method)

Genomic DNA was isolated from leaf and seed material using the Qiagen Plant Mini Kit (Qiagen Inc., Hilden, Germany) following the manufacturer’s protocol. The ITS1–5.8S rDNA–ITS2 region was amplified using the following parameters: initial denaturation at 98 °C for 1 min, 34 cycles of denaturation at 98 °C for 15 s, primer annealing at 55–56 °C for 15 s, and elongation at 72 °C for 30 s, followed by a final elongation at 72 °C for 5 min. Other regions were amplified with the same time parameters; the annealing temperature and primer sequences are presented in Table 4.

### 4.3. Phylogenetic Analysis of the Sequences Obtained by the Sanger Method

The sequence chromatograms were analyzed using Chromas Lite version 2.01 (Technelysium Co. South Brisbane QLD 4101, Brisbane, Australia), and the sequences were then aligned using the Muscle algorithm [52] included in the MEGA XI program [53]. Nuclear and chloroplast genes were analyzed separately due to the widespread hybridization in the Hordeeae tribe and, accordingly, the possible origin of sequences from different genomes from different parents. The best evolutionary models for each region were calculated using jModeltest v. 2.1.10 [54]. Each dataset was then analyzed using the Bayesian method in Mr. Bayes 3.2.2 [55] with 1–2 million iterations; the first 25% of phylogenetic trees were discarded as burn-in. The evolutionary models for ITS sequences were GTR+I+G; for ETS, TIM2+G; for the *trn*L–F region, GTR+G; for *trn*K–*rps*16, TPM2uf; for *psb*A–*trn*H, TVM+I; and for the *ndh*F gene, TVM+I+G. Maximum likelihood analysis was performed using IQ-TREE 2.3.6 [56] with the ultrafast bootstrap option and the same evolutionary models as the Bayesian analysis.

### 4.4. Next-Generation Sequencing

The ITS1 region of rDNA was amplified under the following conditions: initial denaturation at 94 °C for 1 min, followed by 35 cycles of 94 °C for 30 s, 55 °C for 30 s, and 72 °C for 30 s and a final extension at 72 °C for 5 min. We used primers ITS1P [44] and ITS 2 [45]. PCR products were purified using the AMPureXP kit (Beckman Coulter, Indianapolis, IN, USA). Libraries for sequencing were prepared following the user instructions for the MiSeq Reagent Kit Preparation Guide (Illumina) (https://www.illumina.com/products/by-type/sequencingkits/cluster-gen-sequencing-reagents/miseq-reagent-kit-v3.html) (accessed on 15 May 2026). They were sequenced on an Illumina MiSeq instrument (Illumina, San Diego, CA, USA) by the MiSeq^®^ Reagent Kit v3 reagents (600 cycles), paired-end reads (2 × 300), according to the manufacturer’s instructions. The fragments were amplified and sequenced at the Center for Shared Use “Genomic Technologies, Proteomics, and Cell Biology” of the All-Russian Research Institute of Agricultural Microbiology.

### 4.5. Phylogenetic Analysis of NGS Data

The obtained pool of raw sequences was trimmed by Trimmomatic [57] as implemented in Unipro Ugene [58] as follows: PE reads; sliding window trimming with size 4 and quality threshold 12; and minimal read length 130. Then, paired sequences were combined, dereplicated, and sorted by vsearch 2.7.1 [59]. The resulting sequences formed ribotypes in the whole pool of genomic rDNA; they were sorted according to their frequency. For our analysis, we established a threshold of 10 reads per pool of rDNA. The sequences were aligned using MEGA v. 11.0.13 [53]; a ribotype network was built in TCS 1.2.1 [27] by statistical parsimony method and then visualized in TCS BU [28]. In addition, we constructed a phylogenetic tree of the obtained ribotypes by Bayesian and maximum likelihood methods using GTR + G model. Bayesian analysis was conducted with 2–5 millions of generations by Mr. Bayes 3.2.2 [55]. ML analysis was conducted with the aid of IQ-TREE 2.3.6 [56]. In this case, we set a threshold of 40 reads per rDNA pool.

In addition to constructing the phylogenetic diagrams, we examined the ribotype composition of each sample used for NGS. To investigate the ribotype composition of each sample, we generated a minimum spanning tree of the ribotypes within the sample using the p-distance matrix in Arlequin 3.5.1.2 [29]. Then, we summed the percentages of ribotypes relative to the total number of reads in each sample for the largest clusters (sequence groups) of the obtained tree. The ribotype composition of the samples was visualized in Microsoft Excel 16 as pie charts. The resulting charts reflect the ratio of analyzed ribotypes (threshold: 10 reads per rDNA sample) to the total number of reads in each sample.

## 5. Conclusions

When studying this hybrid genus, ×*Agrotrigia*, we observed almost the entire spectrum of post-hybridization changes. In some cases, molecular phylogenetic data clarify the data of comparative morphological studies and even change hypotheses about the origin of hybrid taxa. We confirm the conclusion of N. N. Tzvelev [60] that hybridization allows many highly specialized taxa to de-specialize and continue their evolution to some extent and thereby contributes to an increase in biological diversity on Earth, providing abundant material for natural selection.

## Figures and Tables

**Table 1:** The samples analysed by the Sanger an NGS methods and GenBank sequence accession numbers.

Species	Country of Origin and Location	GenBank Number, ITS	GenBank Number, NGS Data	GenBank Number, ETS	GenBank Number, *trn*K–*rps*16	GenBank Number, *trnL–trnF*	GenBank Number, *trnH–psbA*	GenBank Number, *ndh*F
×*Agrotrigia hajastanica*	Armenia: Shirak Province	PZ492430	PZ523569–PZ523672	PZ457037	PZ497109	PZ457066	PZ652887	PZ568682
×*Agrotrigia hajastanica*	Armenia: Shirak Province	PZ492429		PZ457036	PZ497110	PZ457065	PZ652888	
×*Agrotrigia* sp.	Russia: Stavropol Krai	PZ492427	PZ523346–PZ523391	PZ457038	PZ497101	PZ457068	PZ652908	PZ568683
×*Agrotrigia* sp.	Russia: Stavropol Krai	PZ492428		PZ457039	PZ497102	PZ457067	PZ652907	PZ568684
×*Agrotrigia androssovii*	Turkmenistan: Ashkhabad District	PZ491465	PZ523435–PZ523495	PZ457045	PZ497098	PZ457052	PZ652893	
×*Agrotrigia androssovii*	Russia: Pskov Oblast	PZ492437	PZ523496–PZ523522	PZ457051	PZ497113	PZ457064	PZ652891	PZ568670
×*Agrotrigia kotovii*	Russia: Orenburg Oblast	PZ492436	PZ523523–PZ523568	PZ457048	PZ497111	PZ457063	PZ652889	
×*Agrotrigia kotovii*	Russia: Chelyabinsk Oblast	PZ492435		PZ457049	PZ497112	PZ457062	PZ652890	
*Agropyron desertorum*	Russia: Stavropol Krai	PZ492425	PZ523071–PZ523219	PZ457032	PZ497099	PZ457070	PZ652901	
*Agropyron desertorum*	Russia: Stavropol Krai			PZ457033		PZ457072		
*Agropyron fragile*	Russia: Stavropol Krai			PZ457030		PZ457069	PZ652895	
*Agropyron fragile*	Russia: Stavropol Krai			PZ457031	PZ497100	PZ457071	PZ652896	
*Agropyron imbricatum*	Russia: Stavropol Krai	PZ492423	PZ523220–PZ523345	PZ457034	PZ497096	PZ457074	PZ652900	PZ568680
*Agropyron krylovianum*	Russia: Altai Republic	PZ492433						
*Agropyron pectinatum*	Russia: Stavropol Krai	PZ492424	PZ523406–PZ523434	PZ457035	PZ497097	PZ457073	PZ652894	PZ568681
*Agropyron* sp.	Russia: Altai Krai	PZ492426						
*Elymus caninus*	Russia: Stavropol Krai	PZ492449		PZ457028	PZ497095		PZ652906	
*Elymus caninus*	Russia: Stavropol Krai	PZ492450		PZ457025	PZ497094	PZ457076	PZ652905	
*Elymus caninus*	Russia: Stavropol Krai	PZ492451		PZ457029	PZ497093	PZ457078	PZ652904	PZ568686
*Elymus caninus*	Russia: Stavropol Krai	PZ492452		PZ457027	PZ497092	PZ457077	PZ652903	PZ568685
*Elymus caninus*	Russia: Stavropol Krai	PZ492453		PZ457026	PZ497091	PZ457075	PZ652902	PZ568679
*Elytrigia geniculata*	Russia: Tuva Republic	PZ492434						
*Elytrigia gmelinii*	Russia: Altai Krai	PZ492444						
*Elytrigia intermedia*	Russia: Stavropol Krai	PZ492443	PZ523735–PZ523764	PZ457044	PZ497103	PZ457058	PZ652915	PZ568672
*Elytrigia lolioides*	Russia: Stavropol Krai	PZ492431						
*Elytrigia pulcherrima*	Russia: Stavropol Krai	PZ492445		PZ457043	PZ497106	PZ457060	PZ652912	PZ568673
*Elytrigia pulcherrima*	Russia: Stavropol Krai	PZ492447						
*Elytrigia pulcherrima*	Russia: Stavropol Krai	PZ492446						
*Elytrigia repens*	Russia: Stavropol Krai	PZ492442		PZ457047	PZ497104	PZ457059	PZ652913	PZ568671
*Elytrigia repens*	Russia: Stavropol Krai	PZ492441	PZ523673–PZ523734	PZ457041	PZ497105		PZ652914	
*Elytrigia repens* var. *aristata*	Russia: Stavropol Krai	PZ492439						
*Elytrigia repens* var. *aristata*	Russia: Stavropol Krai	PZ492438	PZ523392–PZ523405	PZ457040		PZ457055	PZ652911	PZ568674
*Elytrigia repens* var. *aristata*	Russia: Altai Republic	PZ492440						
*Elytrigia trichophora*	Russia: Stavropol Krai			PZ457046		PZ457057	PZ652910	
*Elytrigia trichophora* × *E. pulcherrima*	Russia: Stavropol Krai		PZ523765–PZ523799	PZ457042	PZ497107	PZ457056	PZ652909	
*Hordelymus europaeus*	Russia: Stavropol Krai	PZ492454		PZ457024		PZ457054	PZ652897	PZ568678
*Hordeum murinum*	Russia: Stavropol Krai			PZ457023			PZ652917	
*Hordeum vulgare*	Russia: Stavropol Krai	PZ492432		PZ457022			PZ652916	
×*Leymotrigia bergrothii*	Latvia: Riga, Sloka	PZ492448		PZ457050	PZ497114	PZ457061	PZ652892	PZ568675
×*Leymotrigia* sp.	Russia: Altai Republic					PZ457053	PZ652899	PZ568677
×*Leymotrigia* sp.	Russia: Altai Republic				PZ497115		PZ652898	PZ568676
*Secale cereale*	K-10493 (VIR), Afghanistan							PZ568661
*Secale cereale*	K-7985 (VIR), Turkey							PZ568665
*Secale cereale*	K-11754 (VIR), Russia: Omsk Oblast							PZ568667
*Secale cereale*	K-11895 (VIR), Russia: Kirov Oblast							PZ568668
*Secale montanum*	K-9669 (VIR), Azerbaijan							PZ568666
*Secale montanum*	K-10053 (VIR), Armenia							PZ568664
*Secale montanum*	K-10220 (VIR), Azerbaijan							PZ568669
*Secale strictum*	K-2695 (VIR), Italy							PZ568662
*Secale sylvestre*	K-2870 (VIR), Azerbaijan				PZ497090			PZ568663
*Triticosecale rimpaui*								PZ568660

**Table 2:** Major ribotypes (more than 1000 reads per rDNA pool) of the studied species.

Species	Total Number of Reads	Ribotype Symbol (Major Ribotypes)	Number of Reads	% from the Total Number of Reads
×*Agrotrigia hajastanica*	16,199	P1	4891	30
×*Agrotrigia* sp.	8379	P1	1786	21
		P2	1404	10
×*Agrotrigia androssovii* (Turkmenistan)	13,499	St4	3679	27
		P1	1004	7
×*Agrotrigia androssovii* (Pskov Oblast)	11,575	St1	2707	23
		St2	2126	18
		St3	1461	8
×*Agrotrigia kotovii*	11,383	St1	3449	30
		St2	1376	12
*Agropyron desertorum*	26,194	P1	7326	28
		P4	1970	7
*Agropyron imbricatum*	25,703	P1	6563	26
		P2	2574	10
		P3	2220	8
*Agropyron pectinatum*	7702	P1	3888	51
*Elytrigia intermedia*	5538	St4	1096	20
*Elytrigia repens*	25,703	St1	3437	27
		St2	2762	22
*Elytrigia repens* var. *aristata*	6113	St2	1113	18
		St1	1088	18
*Elytrigia trichophora* × *E. pulcherrima*	4633	St6	618	13
		St7	348	8
		St4	333	7
		St5	276	6

**Table 3:** Sequences of the major ribotypes (more than 1000 reads) of the studied species obtained by NGS. The numbers show the position in the alignment of the major ribotypes. D is a deletion.

	1	1	1	1	1	1	1	1	1	1	1	1	1	1	1	1	1	2	2	2	2
	1	1	2	2	2	2	3	3	3	4	4	6	7	7	7	7	7	4	4	7	7
	5	6	6	7	8	9	0	1	3	7	8	1	5	6	7	8	9	2	8	0	1
**P1**	T	C	C	A	T	C	G	T	T	T	T	D	D	D	D	D	T	G	T	C	C
**P2**	C	.	.	.	.	.	.	.	.	.	.	D	D	D	D	D	.	.	.	.	.
**P3**	.	.	.	.	.	.	.	.	.	.	.	D	D	D	D	D	C	.	.	.	.
**P4**	.	.	.	.	.	.	.	.	.	.	.	D	D	D	D	D	.	.	C	.	.
**St1**	G	T	.	.	C	.	.	.	C	G	.	D	T	G	G	G	.	.	.	.	.
**St2**	G	T	.	.	C	.	.	.	C	.	.	D	T	G	G	G	.	.	.	.	.
**St3**	G	T	.	.	C	.	.	.	C	C	.	D	T	G	G	G	.	.	.	.	.
**St4**	G	T	.	.	C	.	.	.	C	A	G	C	T	G	G	G	.	.	.	T	.
**St5**	G	T	.	.	C	.	.	.	C	A	G	C	T	G	G	G	.	A	.	T	T
**St6**	G	T	T	T	.	T	C	A	C	.	G	D	T	G	G	G	.	A	.	T	T
**St7**	G	T	T	T	.	T	C	A	C	.	G	D	T	G	G	G	.	.	.	T	.

**Table 4:** Primers used in the analysis, with their annealing temperature.

Primer Name	Primer Sequence	The Annealing Temperature of the Primers	References
ITS1P	AAC CTT ATC ATT TAG AGG AAG G	55–56 °C	[44]
ITS4	TCC TCC GCT TAT TGA TAT GC		[45]
ETS1fP	TTC CAG TCG GAC GGA TTC	55–56 °C	[46]
ETS-18S-R	AGA CAA GCA TAT GAC TAC TGG CAG G		[47]
rps16_4547_mod	ATC TGG GTT GCT AAC TCA ATG G	55 °C	[20]
trnK-5′r	AAC TAG TCG GAT GGA GTA G		[48]
Tab C	CGA AAT CGG TAG ACG CTA CG	55 °C	[49]
Tab D	GGG GAT AGA GGG ACT TGA AC		
Tab E	GGT TCA AGT CCC TCT ATC CC	50–52 °C	
Tab F	ATT TGA ACT GGT GAC ACG AG		
ndhF-F_mod	AAT CAT TAT TGA CGG TCC AAG ACC	55 °C	[50]
ndhF-R	GGG GGA TAA AAA GAA GGG AAA ATG A		
trnH	CGC GCA TGG TGG ATT CAC AAT CC	55 °C	[51]

## Data Availability

The original contributions presented in this study are included in the article. Further inquiries can be directed to the corresponding authors.
